# Synoviocytes assist in modulating the effect of Ross River virus infection in micromass-cultured primary human chondrocytes

**DOI:** 10.1099/jmm.0.001859

**Published:** 2024-07-19

**Authors:** Wesley Freppel, Elisa X.Y. Lim, Penny A. Rudd, Lara J. Herrero

**Affiliations:** 1 Institute for Biomedicine and Glycomics, Gold Coast Campus, Griffith University, Southport, QLD 4222, Australia

**Keywords:** chondrocyte, fibroblast-like synoviocytes, PCR array, Ross River virus

## Abstract

**Introduction.** Ross River virus (RRV) is a mosquito-borne virus prevalent in Australia and the islands of the South Pacific, where it causes an arthritogenic illness with a hallmark feature of severe joint pain. The joint space is a unique microenvironment that contains cartilage and synovial fluid. Chondrocytes and synoviocytes are crucial components of the joint space and are known targets of RRV infection.

**Hypothesis/Gap statement.** Understanding the relationship between synoviocytes and chondrocytes during RRV infection will provide further insights into RRV-induced joint pathology.

**Methodology.** To better understand the unique dynamics of these cells during RRV infection, we used primary chondrocytes cultured in physiologically relevant micromasses. We then directly infected micromass chondrocytes or infected primary fibroblast-like synoviocytes (FLS), co-cultured with micromass chondrocytes. Micromass cultures and supernatants were collected and analysed for viral load with a PCR array of target genes known to play a role in arthritis.

**Results.** We show that RRV through direct or secondary infection in micromass chondrocytes modulates the expression of cellular factors that likely contribute to joint inflammation and disease pathology, as well as symptoms such as pain. More importantly, while we show that RRV can infect micromass-cultured chondrocytes via FLS infection, FLS themselves affect the regulation of cellular genes known to contribute to arthritis.

**Conclusion.** Single-cell culture systems lack the complexity of *in vivo* systems, and understanding the interaction between cell populations is crucial for deciphering disease pathology, including for the development of effective therapeutic strategies.

## Graphical abstract

A schematic representation of the study methodology. Human micromass-cultured chondrocytes were either directly infected with RRV or secondarily through co-culture with RRV-infected FLS. Micromass chondrocytes were analysed after 1, 3 and 5 days post-infection for gene expression using a PCR array and for viral load using genome copy number qPCR. FLS were analysed for viral load via qPCR, and supernatants were analysed for infectious viral particles using a plaque assay.

## Introduction

Ross River virus (RRV) is a mosquito-borne virus prevalent in Australia and other parts of the South Pacific. RRV infection can lead to a disease characterised by arthralgia, myalgia and arthritis [1,2]. Although RRV infection is not considered life-threatening, it is a significant economic and public health burden causing considerable morbidity [3].

Chondrocytes, the primary cell type found in articular cartilage, are involved in the synthesis and maintenance of the extracellular matrix (ECM), as well as in the expression of cellular factors such as pro-inflammatory cytokines [4]. However, the articular cartilage is non-vascular and depends on the synovial fluid (SF) for nutrient supply [5]. Fibroblast-like synoviocytes (FLS), found in joint-associated tissues, play a critical role in the synthesis of SF, which provides the joint space lubricant and nutrients [6]. Therefore, infection of FLS and the SF could potentially act as a source of infectious virus, which can spread to the cartilage [7,8]. Research on human cases of RRV-induced arthritis revealed inflammation in the synovium [9] and the presence of RRV viral RNA five weeks after symptom onset [10]. While we have already shown that chondrocytes are implicated in RRV pathogenesis as a source of virus replication [11], the role of synoviocytes is still poorly characterised. Additionally, the interplay of the cells during infection is unknown.

The expression of various cellular factors, including cytokines, chemokines and other immune-related molecules, plays a significant role in RRV replication and pathogenesis. Therefore, understanding the impact of FLS in the regulation of cellular factors and responses involved in RRV infection of chondrocytes is crucial for developing effective therapeutic strategies against the virus and for the treatment of joint pain.

*In vitro* culture is often limited to cells growing in two-dimensional monolayers. This is problematic for chondrocytes, which require the expression of key proteins such as collagens and proteoglycans found in the three-dimensional (3D) structure of articular cartilage. In this short study, we used a 3D cell culture model known as ‘micromass’ (MM), where chondrocytes are cultured in a manner to mimic the ultrastructure of the articular cartilage in cell culture [12,13]. This *in vitro* model system retains the ECM architecture and stimulates a better environment for chondrogenic differentiation, making it more physiologically relevant than traditional monolayer cultures. Therefore, using an MM chondrocyte culture system provides a method of studying cartilage *in vitro* without the need for human primary joint/cartilage tissue, which is rarely available [12,13]. We infected MM cultures with RRV either directly or secondarily through co-cultures with RRV-infected FLS. We identified a pool of up- and down-regulated cellular factors in direct RRV-infected MM chondrocytes, many of which are altered when chondrocytes are secondarily infected in co-culture systems with RRV-infected FLS. We identified several genes that likely play a role in RRV-induced joint inflammation and joint pain symptoms. Moreover, we demonstrated that the FLS cells themselves contribute to the gene expression of cellular factors in chondrocytes.

## Methods

### Virus

RRV T48 strain stocks were generated from the pRR64 infectious clone (kindly gifted by Richard Kuhn, Perdue University) and previously described [14].

### Cells

Human primary chondrocytes (Clonetics, Lonza) were cultured in expansion media [low-glucose Dulbecco's Modified Eagle Medium (DMEM) supplemented with 400 µM l-proline, 100 µM ascorbic acid, 10 mM HEPES, GlutaMAX™, 1% minimum essential medium non-essential amino acids (MEM NEAA), 1% penicillin/streptomycin and 10% foetal bovine serum (FBS)] before being cultured as MM at 2 00,000 cells in a droplet of 100 µl [12,13]. Cells were incubated for 3–5 h to allow for attachment and then placed in chondrocyte differentiation media [high-glucose DMEM supplemented with 400 µM l-proline, 100 µM ascorbic acid, 1.25 mg ml^−1^ BSA, 0.1 µM dexamethasone, 10 ng ml^−1^ transforming growth factor-beta 3, 10 mM HEPES, GlutaMAX™, 1x insulin–transferrin–selenium, 1x MEM NEAA and 1x penicillin/streptomycin]. Human FLS (ScienCell, Cat. No. 4700) were cultured in synoviocyte media (ScienCell, Cat. No. 4701) supplemented with 2% FBS (ScienCell, Cat. No. 0010), 1% synoviocyte growth supplement (ScienCell, Cat. No. 4752) and 1% penicillin/streptomycin (ScienCell, Cat. No. 0503).

### RRV infection of MM chondrocytes and FLS

MM chondrocytes were either uninfected with PBS or infected with RRV at MOI 1 for 1 h at 37 °C and then washed twice with PBS prior to the addition of 1 ml of chondrocyte differentiation media.

FLS were cultured on the membrane of cell culture inserts (30,000 cells per insert, 12-well 0.4 µm, SARSTEDT, Cat. No. 83.3931.041). FLS were either uninfected or infected with RRV MOI 1 for 1 h at 37 °C and then washed once with PBS prior to the addition of 0.5 ml of maintenance media (synoviocyte media with reduced serum of 1% FBS). These cell culture inserts were added to the cell culture plates containing the MM chondrocyte culture. Cell culture supernatants were harvested at 0, 1, 2, 3, 4, 5 and 7 dpi, and cells were lysed using a TRIzol (Life Technologies) reagent for isolation of total RNA.

### PCR arrays

Total RNA (1 µg) was reverse transcribed using the Bio-Rad iScript™ cDNA synthesis kit according to the manufacturer’s instructions (Bio-Rad, NSW, Australia). Human Arthritis Tier 1 H96, a predesigned 96-well panel for use with SYBR® Green, was used to screen the expression of 84 genes and performed using a Bio-Rad CFX96 thermal cycler (Bio-Rad) with two-step thermal cycling: 95 °C for 2 min, followed by 40 cycles (95 °C for 5 sec and 60 °C for 30 sec). A melt curve was also performed using 0.5 °C increments ranging between 65 °C and 95 °C. Data were analysed using the comparative ^ΔΔ^Ct method and expressed as fold change in the target gene normalised to the mean of endogenous control gene *hprt1* and relative to the gene expression of the control samples, uninfected chondrocytes alone or uninfected chondrocytes co-cultured with uninfected FLS (normalised such that the controls = 1.0). Data were graphed using conventional heat map representation of two-dimensional tables of numerical data (gene expressions vs. infection conditions), as previously described [15].

### Viral quantification

Infectious particles were determined by plaque assays using Vero cells (ATCC number CCL-81), as previously described [16]. RRV GCN quantification was performed as previously described [17] using SsoAdvanced™ Universal Probes Supermix (Bio-Rad) according to the manufacturer’s instructions. The primer sequences target the RRV nsp3 protein with forward: 5′−CCGTGGCGGGTATTATCAAT−3′; reverse: 5′−AACACTCCCGTCGACAACAGA−3’ and probe: 5’− ATTAAGAGTGTAGCCATCC−3’. The cycling conditions are 95 °C for 15 min, followed by 45 cycles (94 °C for 15 sec and 60 °C for 1 min). Purified plasmid DNA containing the full-length sequence of RRV was serially diluted and used to generate a standard curve for extrapolation of the sample values.

### Statistical analysis

Viral titre and gene expression data from MM chondrocytes are represented by the mean of triplicate samples with sem. Unless otherwise stated, statistical significance was evaluated by multiple comparisons two-way ANOVA with Sidak post-test correction using GraphPad Prism 8.0 software, whereby *P* values <0.05 were considered significant.

## Results

### RRV infects MM-cultured chondrocytes

**Fig. 1.**
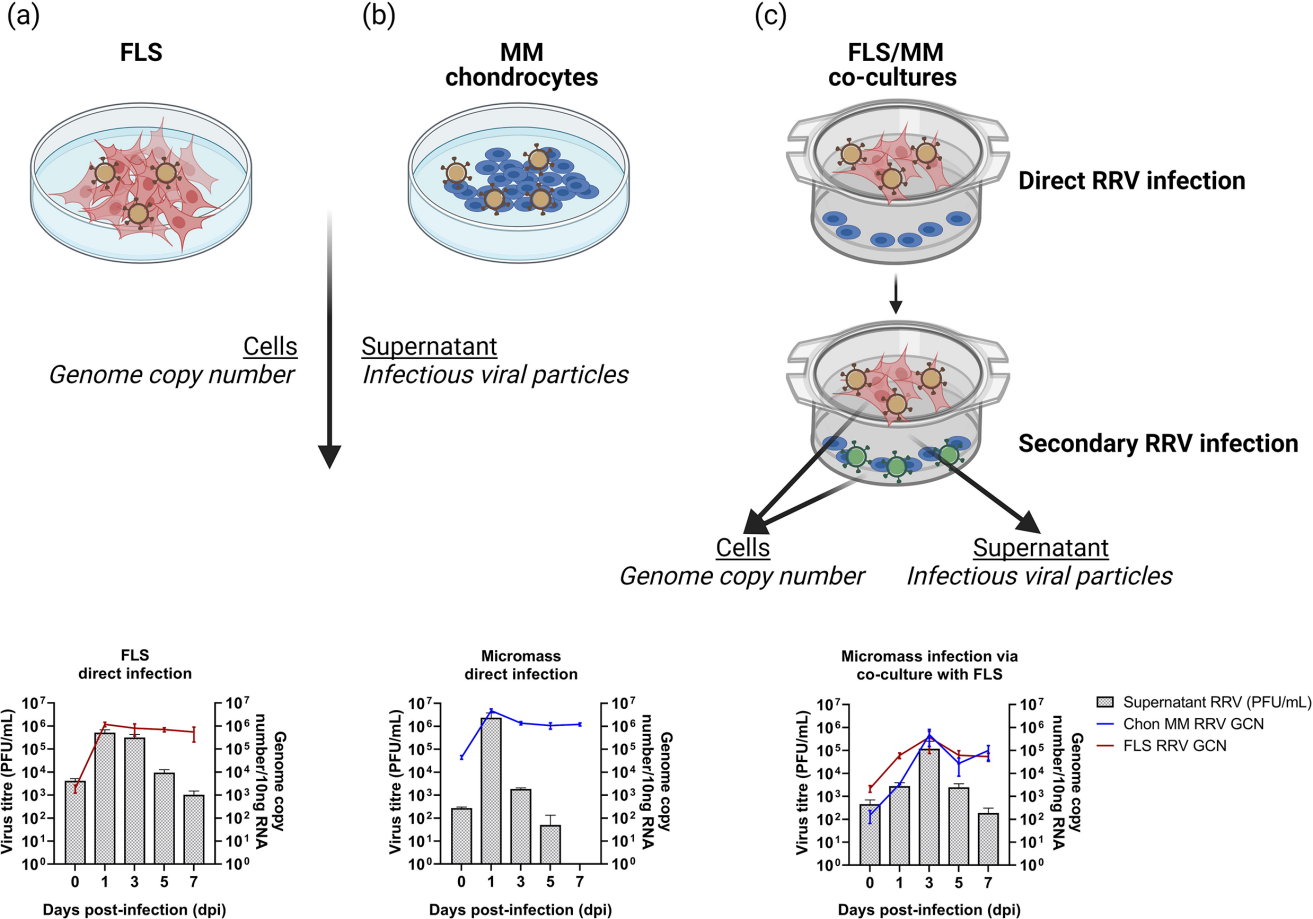
FLS and MM chondrocytes are permissive to RRV infection. Viral titre of RRV were quantified either intracellularly by GCN using quantitative PCR (qPCR) or by infectious viral particles in the supernatant by a plaque assay. Cells and supernatants were collected at 0, 1, 3, 5 and 7 days post-infection. (**a**) RRV-infected FLS (Multiplicity of infection (MOI) = 1). (**b**) RRV-infected MM chondrocytes (MOI = 1). (**c**) RRV-infected FLS/MM chondrocytes (MOI = 1). All Plaque forming units (PFU)/ml and GCN MM alone titres showed significant trends over time. Error bars represent the mean + sem of four wells. Graphs are representative of experiments performed in triplicate.

We show that RRV productively infects chondrocytes when cultured in MM (Fig. 1a) with peak infectious titres at 1 day post-infection (dpi), corresponding to peak intracellular viral genome copy number (GCN). Interestingly, while infectious titres dropped over time, intracellular viral RNA remained high for the 7 day duration of the experiment. Furthermore, RRV infection of FLS peaked at 3 dpi (Fig. 1b) when co-cultured with MM chondrocytes. In line with this observation, secondarily infected MM chondrocytes via FLS in the co-culture system also showed peak titres at 3 dpi.

### MM chondrocyte host genes are differentially regulated by RRV

**Fig. 2.**
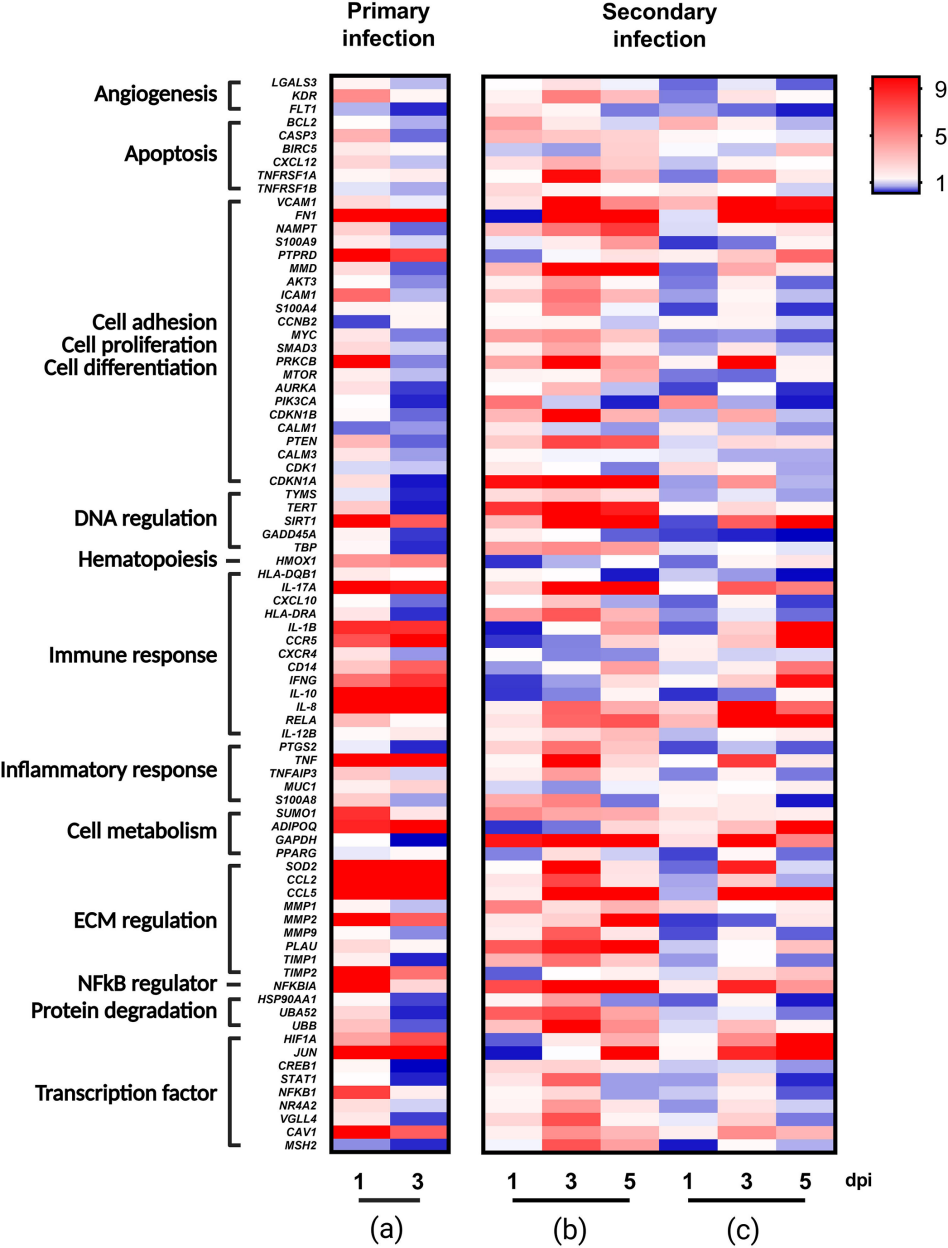
Heat map of cellular factor expressions in MM chondrocytes following RRV infection. (**a**) MM chondrocytes were directly infected with RRV (MOI = 1) for 1 and 3 days. Values were normalised to the housekeeper *hrpt1* gene and expressed relative to control uninfected MM chondrocytes. (**b and c**) MM chondrocytes were secondarily infected through RRV-infected FLS (MOI = 1) for 1, 3 and 5 days. Values were normalised to the housekeeper *HPRT1* gene and expressed relative to control uninfected MM chondrocytes co-cultured with FLS (**b**) or expressed relative to control uninfected MM chondrocytes alone (**c**). Gene up-regulation is represented by a red gradient, while gene down-regulation is represented by a blue gradient. No change in the regulation is represented in white. Genes were grouped based on their primary cellular function.

Our PrimePCR array data show that infection of MM chondrocytes with RRV by direct infection or through co-culture with infected RRV FLS leads to the up-regulation and down-regulation of host genes involved in different aspects of arthritis (Fig. 2).

MM chondrocytes directly infected with RRV and relative to uninfected MM chondrocytes showed a general trend of up-regulating genes involved in immune response and ECM regulation, while genes involved with cell adhesion, proliferation or differentiation and DNA regulation demonstrated a trend for down-regulation (Fig. 2a). When MM was co-cultured with RRV-infected FLS, the genes associated with immune responses and DNA regulation showed opposite modulations relative to uninfected MM co-cultured with uninfected FLS.

### The presence of FLS affects chondrocyte host genes differentially regulated by RRV

To better understand the effect of FLS on RRV-induced MM gene expression, we compared three different settings: (i) the expression of genes from direct RRV infection of MM chondrocytes (Fig. 2a); (ii) the expression of genes in secondary RRV infection of MM chondrocytes relative to uninfected MM chondrocytes co-cultured with uninfected FLS (Fig. 2b) or (iii) relative to uninfected MM chondrocytes alone (Fig. 2c). Interestingly, the presence of FLS in RRV-infected MM chondrocytes resulted in a general trend of down-regulated genes (Fig. 2b).

**Fig. 3.**
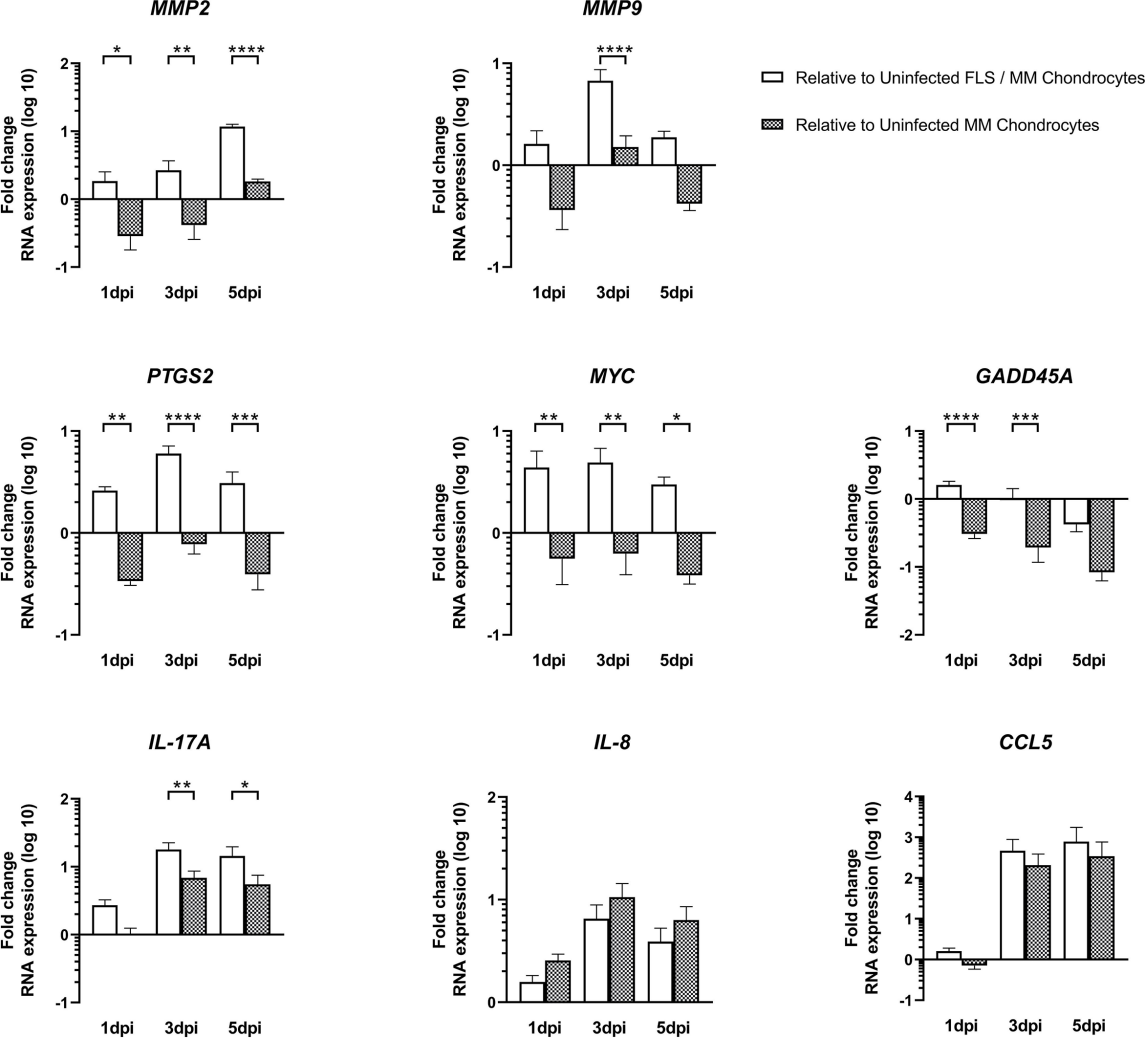
FLS dampens cellular factor gene expression in MM chondrocytes following RRV infection. MM chondrocyte cultures were co-cultured with RRV-infected FLS (MOI of 1) for 1, 3 and 5 dpi. Cells were then analysed for gene expression using a BioRad PrimePCR array. Select genes shown are normalised to *HPRT1* and expressed as log10 fold change relative to uninfected MM alone (grey bars) or uninfected MM co-cultured with uninfected FLS (white bars). Error bars represent the mean + sem of triplicate wells.

We then chose to select genes for further analysis (Fig. 3) and found that chemokine (C-C motif) ligand 5 (*CCL5*), interleukin 17A *(IL-17A)* and 8 (*IL-8)* were consistently up-regulated when directly or secondarily infected with RRV, despite the presence of FLS. Several genes involved in the ECM remodelling such as matrix metalloproteinase 2 (*MMP2*) and 9 (*MMP9*) were up-regulated with RRV infection, but the presence of FLS reversed this trend. The same was observed for genes involved growth arrest and DNA damage-inducible 45A (*GADD45*A: involved in the regulation of DNA repair and cell cycle control), prostaglandin-endoperoxide synthase 2 (*PTGS2*: which mediates responses to physiological stresses including infection and inflammation) and master regulator of cell cycle (*MYC:* a transcription factor that can induce proliferation or apoptosis).

Overall, the results show that the effects on host genes following RRV infection in single MM culture systems are not maintained in co-culture with FLS, suggesting that the co-culture system is dynamic and FLS acts to modulate the effects of infection of MM chondrocytes.

## Discussion

In this short study, we show that RRV can productively infect MM-cultured primary human chondrocytes, either directly or via infection of FLS, resulting in the modulation of genes known to contribute to arthritis.

The results demonstrate that RRV successfully infected chondrocytes cultured in MM with peak infectious titres observed at 1 dpi. Intriguingly, while infectious titres decreased over time, intracellular viral RNA remained high throughout the 7 days of the experiment. These results are in line with our previous study, which demonstrated that human chondrocytes are permissive to RRV *in vivo* and *in vitro* [11]. Interestingly, when FLS were co-cultured with MM chondrocytes, RRV infection peaked on day 3 post-infection. This delay in the peak is probably due to the secondary infection from viral production and viral exocytosis in FLS, followed by the infection of chondrocytes. Overall, these findings suggest that RRV can infect the 3D cell culture models directly or secondarily, leading to productive infection particles.

Our PrimePCR array data revealed that RRV infection, whether through direct or secondary infection, alters the gene expression of host factors known to be associated with arthritis. Not surprisingly, direct RRV infection of MM chondrocytes shows up-regulation of genes associated with immune responses, inflammatory responses, cell metabolism and ECM regulation and a down-regulation trend for genes associated with angiogenesis, cell adhesion, proliferation or differentiation, DNA regulation and protein degradation. Supporting extensive previous *in vitro* studies in a range of cell types, we found significant up-regulation of pro-inflammatory factors with direct RRV infection [11,16, 18]. Pro-inflammatory cytokines are known to contribute to RRV-induced disease in mouse models [19,21] and are frequently found up-regulated in RRV patients [22,26]. Interestingly, we found several pro-inflammatory factors (involved in immune responses, inflammation and ECM degradation) that were consistently up-regulated, regardless of the presence of FLS in cultures, such as IL-17A, IL-8, Tumor necrosis factor (TNF) and CCL5. IL-17A, IL-8 and TNF are involved in inflammation and immune cell recruitment [27,31]. Interestingly, they have been shown to play a role in the development of inflammatory joint pain by increasing the production of other pro-inflammatory cytokines and chemokines [26,32,34]. Moreover, we already reported an increase in TNF during RRV infection [16], suggesting a role for TNF in joint pain during RRV infection. CCL5 is a chemokine that is involved in the recruitment of immune cells to sites of infection or inflammation [35,36]. Previous studies have shown that CCL5 can stimulate the expression and activity of various MMPs, leading to the degradation of collagen and contributing to cartilage destruction [37,38].

Interestingly, not all pro-inflammatory factors remained up-regulated in co-culture systems. Although both Interferon-γ (*IFN*G) and *IL-1B* were up-regulated with direct RRV infection of MM chondrocytes, the inclusion of FLS altered their expression. In co-cultures, MM chondrocyte expression of both *IFNG* and *IL-1B* was initially down-regulated followed by an up-regulation. One possible explanation for this is the delay in peak titre of RRV seen at 3 dpi in MM chondrocytes when secondarily infected via FLS, suggesting that RRV is still the major driver of pro-inflammatory factor expression.

More importantly, our study identifies the up-regulation of ECM factors known to contribute to a multitude of arthritic conditions where pathology is driven by the degradation of cartilage [39,42]. We found that RRV infection altered the gene expression of MMPs and PTGS2, all of which likely contribute to the clinical presentation of RRV infection. MMP2 and MMP9 are enzymes that facilitate the restructuring of the ECM and have been implicated in the pathogenesis of arthritis [43,44]. They have been found to be elevated in the SF and tissue of arthritis patients, contributing to the breakdown of cartilage and bone and promoting disease progression [22,23]. PTGS2 is an enzyme involved in the production of prostaglandins, which are lipid molecules that play a role in inflammation [45,46]. In arthritis, PTGS2 is often up-regulated, leading to increased production of prostaglandins and contributing to the inflammatory response in the affected joints [25]. Inhibiting PTGS2 activity with medications such as nonsteroidal anti-inflammatory drugs can help alleviate the symptoms of arthritis by reducing inflammation and pain [47].

Interestingly, when chondrocytes were infected through RRV-infected FLS, there was a significant loss of the expression of many of the previously identified up-regulated genes, including those involved with ECM regulation and inflammatory response. These data suggest that FLS themselves can impact the gene expression of RRV-infected MM chondrocytes. In fact, in other model systems, it is known that FLS can indirectly impact chondrocytes by producing inflammatory cytokines, which can activate cellular processes and eventually act as a buffer for, or contribute to, cellular stress. In osteoarthritis, for instance, there is an imbalance between the breakdown and repair of cartilage. FLS, under the influence of inflammatory signals, can produce pro-inflammatory molecules and enzymes, such as IL-1ß, TNF, IL-6, IL-15 and IL-18, which contribute to cartilage degradation [48]. In rheumatoid arthritis, the synovial membrane becomes inflamed, leading to an overgrowth of FLS, known as synovial hyperplasia [49]. The excessive proliferation of FLS results in the invasion of the synovium into the articular cartilage and subsequent destruction of the joint. FLS can also activate chondrocytes and promote cartilage degradation through the release of inflammatory mediators and destructive enzymes [50]. In the context of a molecular relationship between FLS and chondrocytes, this crosstalk could perpetuate the destructive cycle seen in RRV-induced arthritis and the chronic pain observed in RRV-induced joint arthralgia.

Conversely, the down-regulation of cellular factors involved, for instance, in DNA regulation or protein degradation during RRV infection could be beneficial for the virus by interfering in molecular pathways and leading to virus-induced arthritis. MYC proteins are transcription factors that play a regulatory role in gene networks and are crucial for self-renewal processes including growth, metabolism and cell cycle progression [51]. In rheumatoid arthritis, there is evidence of increased MYC expression in the synovial tissue [24]. MYC has been implicated in promoting the proliferation of synovial fibroblasts, and studies have shown that increased MYC expression in synovial fibroblasts contributes to their hyperplasia and invasive properties, leading to joint damage [5,52]. Moreover, it has been suggested that MYC can regulate the production of pro-inflammatory cytokines, such as IL-1 and TNF, which are key drivers of inflammation in arthritis [53]. GADD45D is mostly involved in cell cycle regulation, DNA repair and cell growth [54]. It is also known to be induced in response to DNA damage, genotoxic stress and certain cellular signals. While the role of GADD45D in rheumatoid arthritis is not well elucidated, its RNA expression was found to be significantly lower in a cohort of 240 rheumatoid arthritis patients [55]. Moreover, inducing the expression of GADD45 in FLS resulted in the reduction of TNF-induced signalling and decreased expression of MMPs [56], whereas the absence of *GADD45* by knockout worsened K/BxN serum-induced arthritis in mice, leading to a significant increase in JNK pathway signalling, elevated expression of MMP3 and MMP13 genes in the joints and an expansion of the inflammation area [56].

The up-regulation of *CCL5*, *IL-17*A, *IL-8*, *MMP2*, *MMP9*, *PTGS2*, *MYC* and other genes during RRV infection may cause excessive inflammation, leading to tissue damage and may contribute to the symptoms associated with RRV infection, such as joint pain [57]. The down-regulation of *GADD45*D and other genes in response to RRV infection suggests that the virus may be interfering with the normal cellular processes that are necessary for cell growth, survival, DNA regulation and function. This could have several implications for the immune response to RRV infection, including impairing the ability of immune cells to proliferate and respond to the virus. Further research is needed to fully understand the roles of these genes and proteins in the pathogenesis of RRV infection and to develop targeted therapies to mitigate the symptoms associated with the disease.

While the MM chondrocyte model used in this study mimics the ultrastructure of the articular cartilage in cell culture, there are still limitations in our model, which cannot fully replicate the cellular and molecular activities of a complex joint *in vivo*. Such a complex joint system would include cells such as macrophages, dendritic cells, mast cells and T cells, all of which could have an impact on the expression of pro-inflammatory cytokines involved in cartilage destruction from chondrocytes and FLS. Overall, with this study, we offer the first analysis of gene expression in a 3D cell culture of chondrocytes co-cultured with FLS. The relationship between FLS and chondrocytes may play an important role in the pathogenesis of inflammatory joint symptoms caused by alphaviruses, and further research is needed to fully understand the mechanisms by which RRV and other alphaviruses lead to the up- and down-regulation of cellular factors in these cell types *in vitro* and *in vivo*.

## Abbreviations

ccl5: chemokine (C-C motif) ligand 5
3D: three dimensional
DMEM: Dulbecco's Modified Eagle Medium
dpi: days post-infection
ECM: extracellular matrix
FBS: foetal bovine serum
FLS: fibroblast-like synoviocytes
GCN: genome copy number
MEM NEAA: minimum essential medium non-essential amino acids
MM: micromass
mmp: matrix metalloproteinase
qPCR: quantitative PCR
RRV: Ross River virus
SF: synovial fluid
